# Asparagine reduces the risk of schizophrenia: a bidirectional two-sample mendelian randomization study of aspartate, asparagine and schizophrenia

**DOI:** 10.1186/s12888-024-05765-5

**Published:** 2024-04-19

**Authors:** Huang-Hui Liu, Yao Gao, Dan Xu, Xin-Zhe Du, Si-Meng Wei, Jian-Zhen Hu, Yong Xu, Liu Sha

**Affiliations:** 1 Department of Psychiatry, First Hospital/First Clinical Medical College of Shanxi Medical University, NO.85 Jiefang Nan Road, Taiyuan, China; 2 Shanxi Key Laboratory of Artificial Intelligence Assisted Diagnosis and Treatment for Mental Disorder, First Hospital of Shanxi Medical University, Taiyuan, China

**Keywords:** Aspartate, Asparagine, Schizophrenia, Mendelian randomization

## Abstract

**Background:**

Despite ongoing research, the underlying causes of schizophrenia remain unclear. Aspartate and asparagine, essential amino acids, have been linked to schizophrenia in recent studies, but their causal relationship is still unclear. This study used a bidirectional two-sample Mendelian randomization (MR) method to explore the causal relationship between aspartate and asparagine with schizophrenia.

**Methods:**

This study employed summary data from genome-wide association studies (GWAS) conducted on European populations to examine the correlation between aspartate and asparagine with schizophrenia. In order to investigate the causal effects of aspartate and asparagine on schizophrenia, this study conducted a two-sample bidirectional MR analysis using genetic factors as instrumental variables.

**Results:**

No causal relationship was found between aspartate and schizophrenia, with an odds ratio (OR) of 1.221 (95%CI: 0.483–3.088, *P*-value = 0.674). Reverse MR analysis also indicated that no causal effects were found between schizophrenia and aspartate, with an OR of 0.999 (95%CI: 0.987–1.010, *P*-value = 0.841). There is a negative causal relationship between asparagine and schizophrenia, with an OR of 0.485 (95%CI: 0.262-0.900, *P*-value = 0.020). Reverse MR analysis indicates that there is no causal effect between schizophrenia and asparagine, with an OR of 1.005(95%CI: 0.999–1.011, *P*-value = 0.132).

**Conclusion:**

This study suggests that there may be a potential risk reduction for schizophrenia with increased levels of asparagine, while also indicating the absence of a causal link between elevated or diminished levels of asparagine in individuals diagnosed with schizophrenia. There is no potential causal relationship between aspartate and schizophrenia, whether prospective or reverse MR. However, it is important to note that these associations necessitate additional research for further validation.

## Introduction

 Schizophrenia is a serious psychiatric illness that affects 0.5 -1% of the global population [1]. The burden of mental illness was estimated to account for 7% of all diseases worldwide in 2016, and nearly 20% of all years lived with disability [2]. The characteristics of schizophrenia are positive symptoms, negative symptoms, and cognitive symptoms, which are often severe functional impairments and significant social maladaptations for patients suffering from schizophrenia [3]. It is still unclear what causes schizophrenia and what the pathogenesis is. There are a number of hypotheses based on neurochemical mechanisms, including dopaminergic and glutamatergic systems [4]. Although schizophrenia research has made significant advances in the past, further insight into its mechanisms and causes is still needed.

Association genetics research and genome-wide association studies have successfully identified more than 24 candidate genes that serve as molecular biomarkers for the susceptibility to treatment- refractory schizophrenia (TRS). It is worth noting that some proteins in these genes are related to glutamate transfer, especially the N-methyl-D-aspartate receptor (NMDAR) [5]. It is thought that NMDARs are important for neural plasticity, which is the ability of the brain itself to adapt to new environments. With age, NMDAR function usually declines, which may lead to decreased plasticity, leading to learning and memory problems. Consequently, the manifestation of cognitive deficits observed in diverse pathologies, including Alzheimer’s disease, amyotrophic lateral sclerosis, Huntington’s disease, Parkinson’s disease, schizophrenia, and major depression, can be attributed to the dysfunction of NMDAR [4]. There are two enantiomers of aspartate (Asp): L and D [6]. In the brain, D-aspartate (D-Asp) stimulates glutamate receptors and dopaminergic neurons through its direct NMDAR agonist action [7]. According to the glutamate theory of Sch, glutamate NMDAR dysfunction is a primary contributor to the development of this psychiatric disorder and TRS [8]. It has been shown in two autopsy studies that D-Asp of prefrontal cortex neurons in patients with schizophrenia are significantly reduced, which is related to an increased expression of D-Asp oxidase [9] or an increased activity of D-Asp oxidase [10]. Several studies in animal models and humans have shown that D-amino acids, particularly D-Ser and D-Asp [11], are able to modulate several NMDAR-dependent processes, including synaptic plasticity, brain development, cognition and brain ageing [12]. In addition, D-Asp is synthesized in hippocampal and prefrontal cortex neurons, which play an important role in the development of schizophrenia [13]. It has been reported that the precursor substance of asparagine (Asn), aspartate, activates the N-methyl-D-aspartate receptor [14]. Asparagine is essential for the survival of all cells [15], and it was decreased in schizophrenia patients [16]. Asparagine can cause metabolic disorders of alanine, aspartate, and glutamic acid, leading to dysfunction of the glutamine-glutamate cycle and further affecting it Gamma-Aminobutyric Acid(GABA) level [17].It is widely understood that the imbalance of GABA levels and NMDAR plays a crucial role in the pathogenesis of schizophrenia, causing neurotoxic effects, synaptic dysfunction, and cognitive impairments [18].Schizophrenic patients exhibited significantly higher levels of serum aspartate, glutamate, isoleucine, histidine and tyrosine and significantly lower concentrations of serum asparagine, tryptophan and serine [19]. Other studies have also shown that schizophrenics have higher levels of asparagine, phenylalanine, and cystine, and lower ratios of tyrosine, tryptophan, and tryptophan to competing amino acids, compared to healthy individuals [20]. Aspartate and asparagine’s association with schizophrenia is not fully understood, and their causal relationship remains unclear.

The MR method is a method that uses Mendelian independence principle to infer causality, which uses genetic variation to study the impact of exposure on outcomes. By using this approach, confounding factors in general research are overcome, and causal reasoning is provided on a reasonable temporal basis [21]. The instrumental variables for genetic variation that are chosen must adhere to three primary hypotheses: the correlation hypothesis, which posits a robust correlation between single nucleotide polymorphisms (SNPs) and exposure factors; the independence hypothesis, which asserts that SNPs are not affected by various confounding factors; the exclusivity hypothesis, which maintains that SNPs solely influence outcomes through on exposure factors. In a recent study, Mendelian randomization was used to reveal a causal connection between thyroid function and schizophrenia [22]. According to another Mendelian randomization study, physical activity is causally related to schizophrenia [23]. Therefore, this study used Mendelian randomization method to explore the causal effects of aspartate on schizophrenia and asparagine on schizophrenia.

To elucidate the causal effects of aspartate and asparagine on schizophrenia. This study used bidirectional MR analysis. In the prospective analysis of MR, the exposure factors under consideration were aspartate and asparagine, while the outcome of interest was the risk of schizophrenia. On the contrary, in the reverse MR analysis, schizophrenia was utilized as the exposure factor, with aspartate and asparagine being chosen as the outcomes.

## Materials and methods

### Obtaining data sources

#### Select genetic tools closely related to aspartate or asparagine

In this research, publicly accessible GWAS summary statistical datasets from the MR basic platform were utilized. These datasets consisted of 7721 individuals of European ancestry [24] for the exposure phenotype instrumental variable of aspartate, and 7761 individuals of European ancestry [24] for the exposure phenotype instrumental variable of asparagine.

#### Select genetic tools closely related to schizophrenia

Data from the MR basic platform was used in this study for GWAS summary statistics, which included 77,096 individuals of European ancestry [5], as instrumental variables related to schizophrenia exposure phenotype.

#### Obtaining result data

The publicly accessible GWAS summary statistical dataset for schizophrenia was utilized on the MR basic platform, with a sample size of 77096. Additionally, the summary level data for aspartate and asparagine were obtained from the publicly available GWAS summary dataset on the MR basic platform, with sample sizes of 7721 and 7761, respectively, serving as outcome variables.

### Instrumental variable filtering

#### Eliminating linkage disequilibrium

The selection criteria for identifying exposure related SNPs from the aggregated data of GWAS include: (1) Reaching a significance level that meets the threshold for whole genome research, expressed as *P*-value < 5 * 10 − 6 [25]; (2) Ensure the independence of the selected SNPs and eliminate linkage disequilibrium SNPs (*r*^2^ < 0.001, window size of 10000KB) [26]; (3) There are corresponding data related to the research results in the GWAS summary data.

#### Eliminating weak instruments

To evaluate whether the instrumental variables selected for this MR study have weak values, we calculated the F-statistic. If the F-value is greater than 10, it indicates that there are no weak instruments in this study, indicating the reliability of the study. Using the formula F =[(N-K-1)/K] × [R^2^/(1-R^2^)], where N denotes the sample size pertaining to the exposure factor, K signifies the count of instrumental variables, and R^2^ denotes the proportion of variations in the exposure factor that can be elucidated by the instrumental variables.

#### The final instrumental variable obtained

As a result of removing linkage disequilibrium and weak instrumental variables, finally, 3 SNPs related to aspartate and 24 SNPs related to asparagine were selected for MR analysis.

### Bidirectional MR analysis

#### Research design

**Fig. 1:**
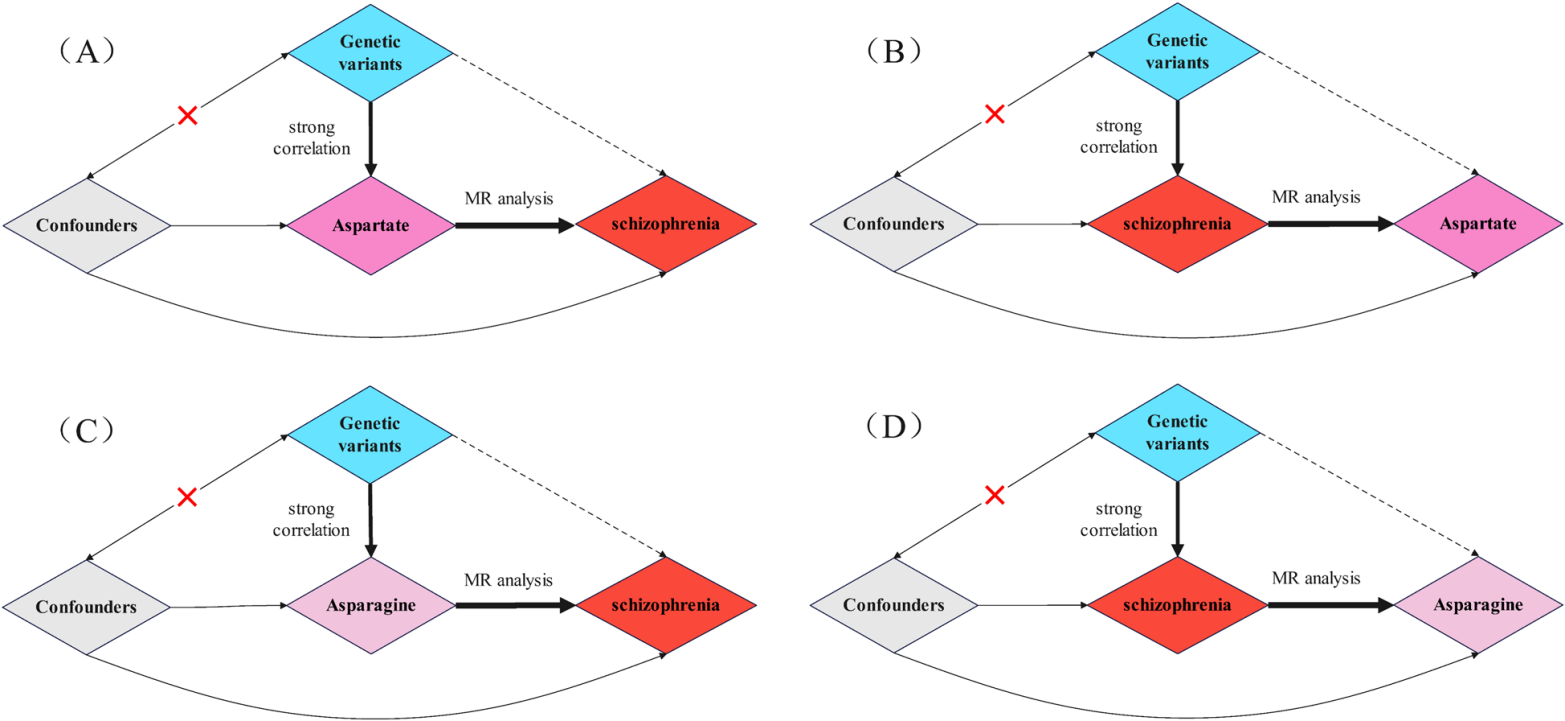
**A** MR analysis of aspartate and schizophrenia (located in the upper left corner). **B **MR analysis of schizophrenia and aspartate (located in the upper right corner). **C **MR analysis of asparagine and schizophrenia (located in the lower left corner). **D **MR analysis of schizophrenia and asparagine (located in the lower right corner)

Figure 1 presents a comprehensive depiction of the overarching design employed in the MR analysis undertaken in this study. We ascertained SNPs exhibiting robust correlation with the target exposure through analysis of publicly available published data, subsequently investigating the existence of a causal association between these SNPs and the corresponding outcomes. This study conducted two bidirectional MR analyses, one prospective and reverse MR on the causal relationship between aspartate and schizophrenia, and the other prospective and reverse MR on the causal relationship between asparagine and schizophrenia.

#### Statistical analysis

Weighted median, weighted mode, MR Egger, and inverse variance weighting (IVW) were used to conduct a MR study. The primary research findings were derived from the results obtained through IVW, the results of sensitivity analysis using other methods to estimate causal effects are considered. Statistical significance was determined if the *P*-value was less than 0.05. To enhance the interpretation of the findings, this study converted the beta values obtained in to OR, accompanied by the calculation of a 95% confidence interval (CI).

#### Test for directional horizontal pleiotropy

This study used MR Egger intercept to test horizontal pleiotropy. If the *P*-value is greater than 0.05, it indicates that there is no horizontal pleiotropy in this study, meaning that instrumental variables can only regulate outcome variables through exposure factors.

## Results

### Results of bidirectional MR analysis of aspartate and schizophrenia

#### Analysis results of aspartate and schizophrenia

**Fig. 2:**
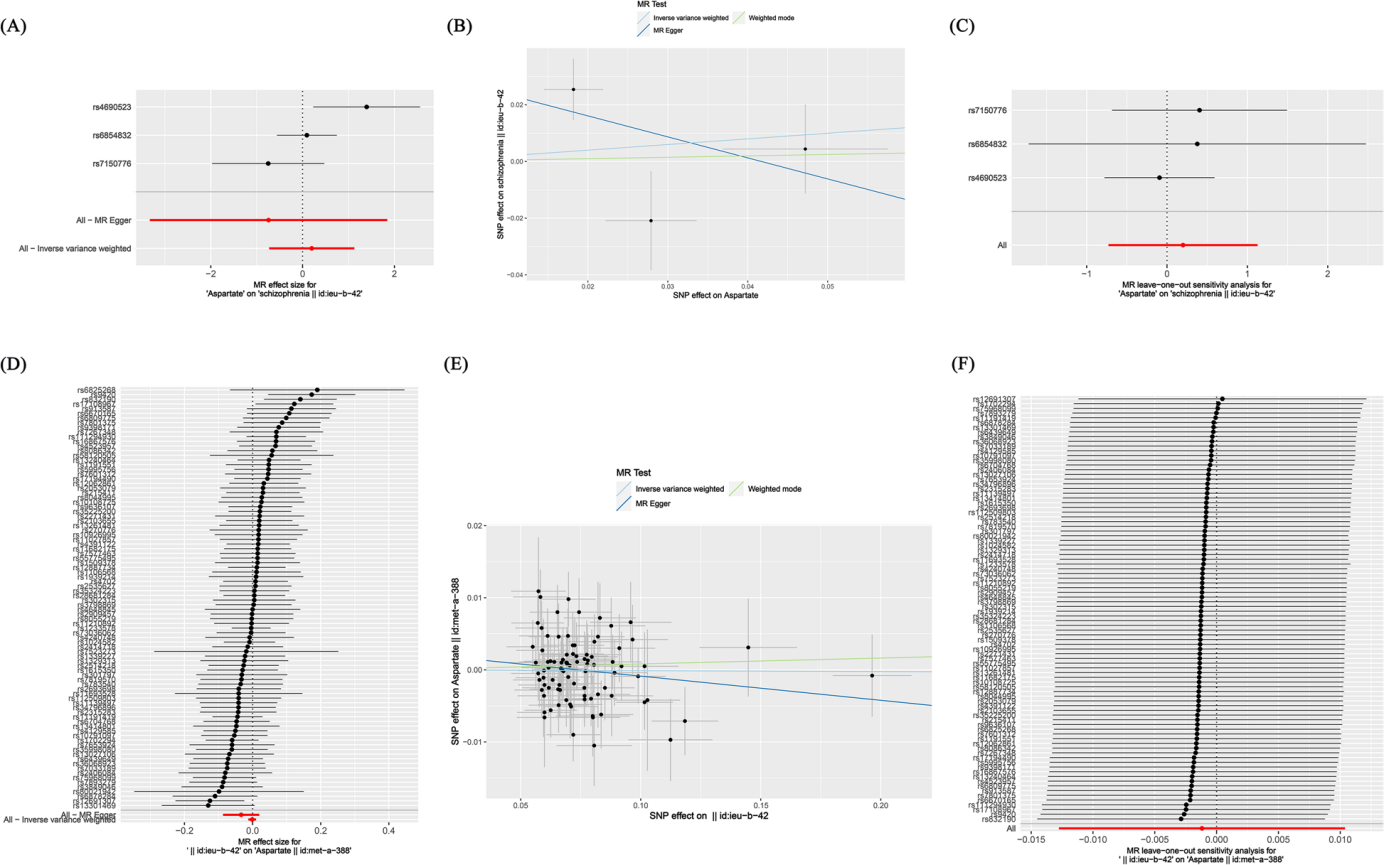
Depicts the causal association between aspartate and schizophrenia through diverse statistical analyses, as well as the causal association between schizophrenia and aspartate through diverse statistical analyses. **A** The forest plot of aspartate related SNPs and schizophrenia analysis results, with the red line showing the MR Egger test and IVW method. **B **Scatter plot of the analysis results of aspartate and schizophrenia, with the slope indicating the strength of the causal relationship. **C **Leave-one-out test of research results on aspartate and schizophrenia. **D** The forest plot of schizophrenia related SNPs and aspartate analysis results, with the red line showing the MR Egger test and IVW method. **E **Scatter plot of the analysis results of schizophrenia and aspartate, with the slope indicating the strength of the causal relationship. **F **Leave-one-out test of research results on schizophrenia and aspartate

In prospective MR analysis, this study set aspartate as the exposure factor and schizophrenia as the outcome. We used 3 SNPs significantly associated with aspartate screened across the entire genome. The instrumental variables exhibited F-values exceeding 10, signifying the absence of weak instruments and thereby affirming the robustness of our findings. Through MR analysis (Fig. 2A), we assessed the individual influence of each SNP locus on schizophrenia. The results of the IVW method indicate that no causal effect was found between aspartate and schizophrenia, with an OR of 1.221 (95%CI: 0.483–3.088, *P*-value = 0.674).

In addition, the analyses conducted using the weighted mode and weighted median methods yielded similar results, indicating the absence of a causal association between aspartate and schizophrenia. Furthermore, the MR Egger analysis demonstrated no statistically significant disparity in effectiveness between aspartate and schizophrenia, as evidenced by a *P*-value greater than 0.05 (Table 1; Fig. 2B).

In order to test the reliability of the research results, this study used MR Egger intercept analysis to examine horizontal pleiotropy, and the result was *P*-value = 0.579 > 0.05, indicating the absence of level pleiotropy. Furthermore, a leave-one-out test was conducted to demonstrate that no single SNP had a substantial impact on the stability of the results, indicating that this study has considerable stability (Fig. 2C). Accordingly, the MR analysis results demonstrate the conclusion that aspartate and schizophrenia do not exhibit a causal relationship.

#### Analysis results of schizophrenia and aspartate

Different from prospective MR studies, in reverse MR studies, schizophrenia was set as an exposure factor and aspartate was set as the outcome. Through MR analysis (Fig. 2D), we assessed the individual influence of each SNP locus on aspartate .The results of the IVW method indicate that there is no causal effect between schizophrenia and aspartate, with an OR of 0.999(95%CI: 0.987–1.010, *P*-value = 0.841). Similarly, the weighted mode, weighted median methods also failed to demonstrate a causal link between schizophrenia and aspartate. Additionally, the MR Egger analysis did not reveal any statistically significant difference in effectiveness between schizophrenia and aspartate (*P*-value > 0.05) (Table 1 and Fig .2E).

The MR Egger intercept was used to test horizontal pleiotropy, and the result was *P*-value = 0.226 > 0.05, proving that this study is not affected by horizontal pleiotropy. Furthermore, a leave-one-out test revealed that no individual SNP significantly influenced the robustness of the findings (Fig. 2F).

### Results of bidirectional MR analysis of asparagine and schizophrenia

#### Analysis results of asparagine and schizophrenia

**Fig. 3:**
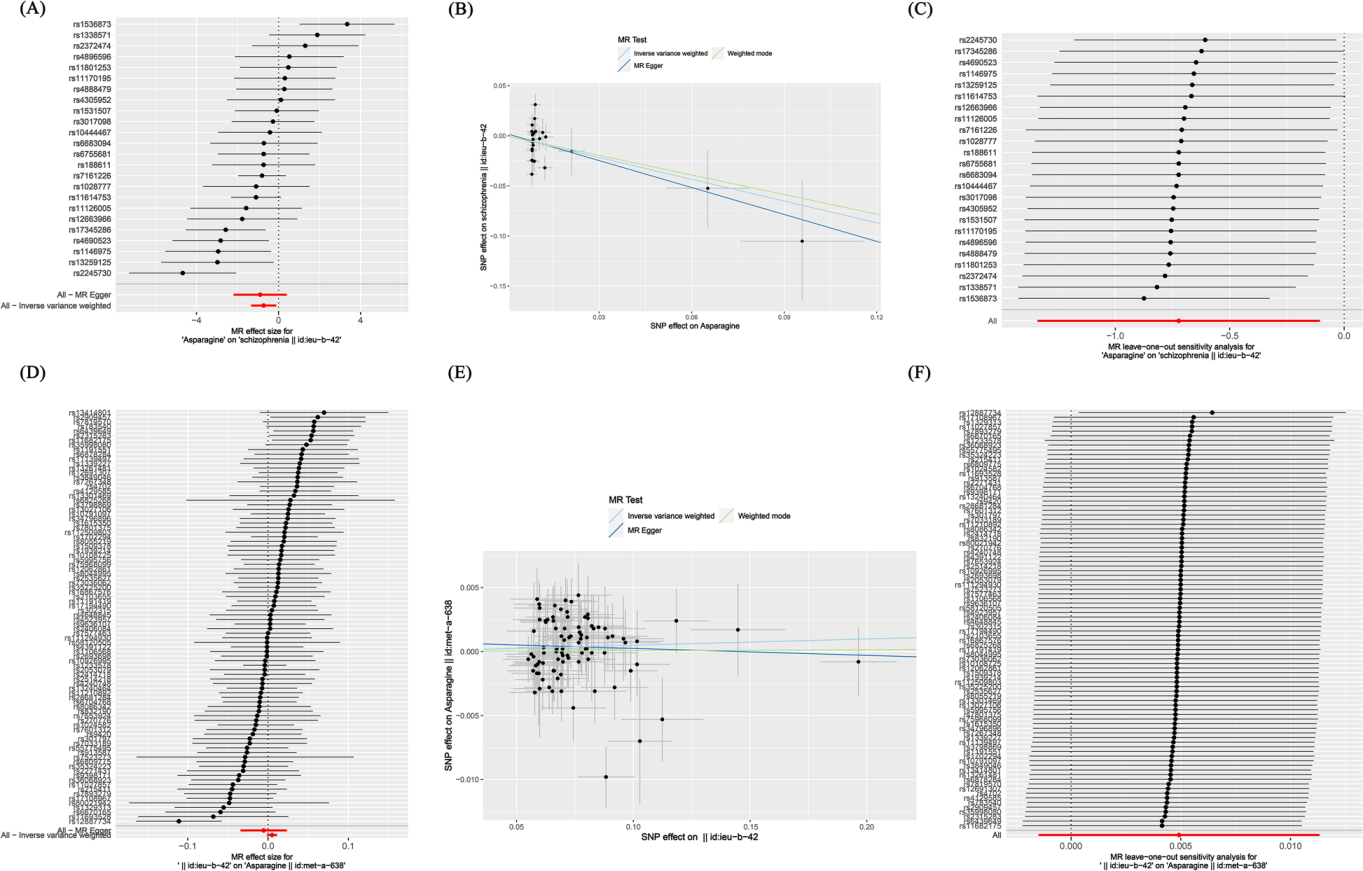
Depicts the causal association between asparagine and schizophrenia through diverse statistical analyses, as well as the causal association between schizophrenia and asparagine through diverse statistical analyses. **A **The forest plot of asparagine related SNPs and schizophrenia analysis results, with the red line showing the MR Egger test and IVW method. **B **Scatter plot of the analysis results of asparagine and schizophrenia, with the slope indicating the strength of the causal relationship. **C** Leave-one-out test of research results on asparagine and schizophrenia. **D** The forest plot of schizophrenia related SNPs and asparagine analysis results, with the red line showing the MR Egger test and IVW method. **E** Scatter plot of the analysis results of schizophrenia and asparagine, with the slope indicating the strength of the causal relationship. **F** Leave-one-out test of research results on schizophrenia and asparagine

In prospective MR studies, we used asparagine as an exposure factor and schizophrenia as a result to investigate the potential causal relationship between them. Through a rigorous screening process, we identified 24 genome-wide significant SNPs associated with asparagine. In addition, the instrumental variable F values all exceeded 10, indicating that this study was not affected by weak instruments, thus proving the stability of the results. This study conducted MR analysis to evaluate the impact of all SNP loci on schizophrenia. (Fig. 3A). According to the results of IVW, a causal relationship was found between asparagine and schizophrenia, and the relationship is negatively correlated, with an OR of 0.485 (95%CI: 0.262-0.900, *P*-value = 0.020).

The weighted median results also showed a causal relationship between asparagine and schizophrenia, and it was negatively correlated. In the weighted mode method, asparagine and schizophrenia did not have a causal relationship, while in the MR Egger method, there was no statistically significant difference in efficacy between them (*P*-value > 0.05) (Table 1; Fig. 3B).

In order to examine the horizontal pleiotropy, the MR Egger intercept was applied, and *P*-value = 0.768 > 0.05 result proves that this study is not affected by horizontal pleiotropy Furthermore, a leave-one-out test was conducted to demonstrate that no individual SNP had a substantial impact on the stability of the results, indicating that this study has good stability. (Fig. 3C). Therefore, MR analysis shows that asparagine is inversely proportional to schizophrenia.

#### Analysis results of schizophrenia and asparagine

In reverse MR analysis, schizophrenia is considered an exposure factor, and asparagine is considered the result, studying the causal effects of schizophrenia and asparagine. Through MR analysis (Fig. 3D), we assessed the individual influence of each SNP locus on s asparagine. The IVW method results indicated no potential causal relationship between schizophrenia and asparagine, with an OR of 1.005(95%CI: 0.999–1.011, *P*-value = 0.132). The research results of weighted mode method and weighted median method did not find a causal effects of schizophrenia and asparagine. Additionally, the MR Egger analysis did not reveal any statistically significant difference in effectiveness between schizophrenia and asparagine (*P*-value > 0.05) (Table 1; Fig. 3E).

In order to examine the horizontal pleiotropy, the MR Egger intercept was applied, and the result was *P*-value = 0.474 > 0.05, proving that this study is not affected by horizontal pleiotropy. Furthermore, a leave-one-out test was conducted to demonstrate that no individual SNP had a substantial impact on the stability of the results, indicating that this study has good stability (Fig. 3F).

**Table 1:** Results of various analytical methods

Exposure	Outcome	Method	Nsnp	OR		Se	P
Aspartate	Schizophrenia	MR Egger	3	0.477	1.320		0.674
		Weighted median	3	1.089	0.338		0.802
		IVW	3	1.221	0.474		0.674
		Weighted mode Egger intercept	3	1.050	0.376 0.04		0.907 0.579
Schizophrenia	Aspartate	MR Egger	90	0.997	0.021		0.221
		Weighted median	90	1.002	0.007		0.785
		IVW	90	0.999	0.005		0.841
		Weighted mode Egger intercept	90	0.993	0.008 0.002		0.672 0.226
Asparagine	Schizophrenia	MR Egger	24	0.408	0.664		0.191
		Weighted median	24	0.478	0.334		0.027
		IVW	24	0.485	0.315		0.022
		Weighted mode Egger intercept	24	0.522	0.428 0.007		0.142 0.768
Schizophrenia	Asparagine	MR Egger	90	0.995	0.015		0.714
		Weighted median	90	1.003	0.004		0.491
		IVW	90	1.005	0.003		0.132
		Weighted mode Egger intercept	90	1.001	0.009 0.001		0.941 0.474

*Nsnp* Number of SNPs, *OR* odds ratio, *PP- value*

## Discussion

**Fig. 4:**
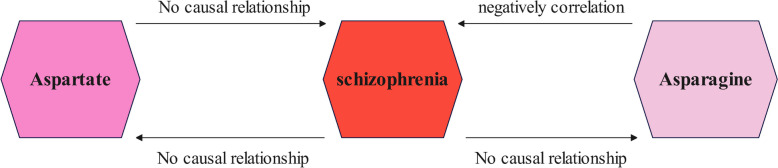
Summary of results from bidirectional two-sample MR study

In this study, the MR analysis results after sensitivity analysis suggested a causal relationship between asparagine and schizophrenia, which was negatively correlated. However, the reverse MR analysis did not reveal any potential relationship between schizophrenia and asparagine, no potential causal relationship between aspartate and schizophrenia was found in both prospective and reverse MR analyses (Fig. 4).

The levels of asparagine in schizophrenia patients decrease, according to studies [16]. Based on the findings of the Madis Parksepp research team, a continuous five-year administration of antipsychotic drugs (AP) has been observed to induce significant metabolic changes in individuals diagnosed with schizophrenia. Significantly, the concentrations of asparagine, glutamine (Gln), methionine, ornithine, and taurine have experienced a substantial rise, whereas aspartate, glutamate (Glu), and alpha-aminoadipic acid(α-AAA) levels have demonstrated a notable decline. Olanzapine (OLZ) treatment resulted in significantly lower levels of Asn compared to control mice [27]. Asn and Asp play significant roles in various biological processes within the human body, such as participating in glycoprotein synthesis and contributing to brain functionality. It is worth noting that the ammonia produced in brain tissue needs to have a rapid excretion pathway in the brain. Asn plays a crucial role in regulating cellular function within neural tissues through metabolic control. This amino acid is synthesized by the combination of Asp and ammonia, facilitated by the enzyme asparagine synthase. Additionally, the brain effectively manages ammonia elimination by producing glutamine Gln and Asn. This may be an explanation for the significant increase in Asn and Gln levels (as well as a decrease in Asp and Glu levels) during 5 years of illness and after receiving AP treatment [28]. The study by Marie Luise Rao’s team compared unmedicated schizophrenic patients, healthy individuals and patients receiving antipsychotic treatment. Unmedicated schizophrenics had higher levels of asparagine, citrulline, phenylalanine, and cysteine, while the ratios of tyrosine, tryptophan, and tryptophan to competing amino acids were significantly lower than in healthy individuals [29].

The findings of our study demonstrate an inverse association between asparagine levels and the susceptibility to schizophrenia, suggesting that asparagine may serve as a protective factor against the development of this psychiatric disorder. However, we did not find a causal relationship between schizophrenia and asparagine. Consequently, additional investigation and scholarly discourse are warranted to gain a comprehensive understanding of this complex association.

Two different autopsy studies measured D-ASP levels in two different brain samples from patients with schizophrenia and a control group [14]. The first study, which utilized a limited sample size (7–10 subjects per diagnosis), demonstrated a reduction in D-ASP levels within the prefrontal cortex (PFC) postmortem among individuals diagnosed with schizophrenia, amounting to approximately 101%. This decrease was found to be correlated with a notable elevation in D-aspartate oxidase (DDO) mRNA levels within the same cerebral region [30]. In addition, the second study was conducted on a large sample size (20 subjects/diagnosis/brain regions). The findings of this study indicated a noteworthy decrease of approximately 30% in D-ASP selectivity within the dorsal lateral PFC (DLPFC) of individuals diagnosed with schizophrenia, when compared to corresponding brain regions of individuals without schizophrenia. However, no significant reduction in D-ASP was observed in the hippocampus of patients with schizophrenia. The decrease in D-Asp content was associated with a significant increase (about 25%) in DDO enzyme activity in the DLPFC of schizophrenia patients. This observation highlights the existence of a dysfunctional metabolic process in DDO activity levels in the brains of schizophrenia patients [31].

Numerous preclinical investigations have demonstrated the influence of D-Asp on various phenotypes reliant on NMDAR, which are linked to schizophrenia. After administering D-Asp to D-Asp oxidase gene knockout mice, the abnormal neuronal pre-pulse inhibition induced by psychoactive drugs such as MK-801 and amphetamine was significantly reduced by the sustained increase in D-Asp [32]. According to a review, free amino acids, specifically D-Asp and D-Ser (D-serine), have been identified as highly effective and safe nutrients for promoting mental well-being. These amino acids not only serve as integral components of the central nervous system’s structural proteins, but also play a vital role in maintaining optimal functioning of the central nervous system. This is due to their essential role in regulating neurotransmitter levels, including dopamine, norepinephrine, serotonin, and others. For many patients with schizophrenia, a most persistent and effective improvement therapy may be supplementing amino acids, which can improve the expected therapeutic effect of AP and alleviate positive and negative symptoms of schizophrenia [33].

Numerous studies have demonstrated a plausible correlation between aspartate and schizophrenia; however, our prospective and reverse MR investigations have failed to establish a causal link between aspartate and schizophrenia. This discrepancy may be attributed to the indirect influence of aspartate on the central nervous system through the stimulation of NMDAR, necessitating further investigation to elucidate the direct relationship between aspartate and schizophrenia.

This study used a bidirectional two-sample MR analysis method to explore the causal relationship between aspartate and asparagine with schizophrenia, as well as its inverse relationship [34]. The utilization of MR analysis presents numerous benefits in the determination of causality [35]. Notably, the random allocation of alleles to gametes within this method permits the assumption of no correlation between instrumental variables and confounding factors. Consequently, this approach effectively alleviates bias stemming from confounding factors during the inference of causality. Furthermore, the study’s utilization of a substantial sample size in the GWAS summary data engenders a heightened level of confidence in the obtained results [36]. Consequently, this investigation not only advances the existing body of research on the relationship between aspartate and asparagine with schizophrenia, but also contributed to clinical treatment decisions for patients with schizophrenia.

Nevertheless, this study possesses certain limitations, as it solely relies on populations of European ancestry for both exposure and results. Consequently, it remains uncertain whether these findings can be replicated among non-European races, necessitating further investigation. In addition, in this study, whether the effects of aspartate and asparagine on schizophrenia vary by gender or age cannot be evaluated, and stratified MR analysis should be performed. Additional experimental research is imperative for a comprehensive understanding of the underlying biological mechanisms connecting aspartate and asparagine with schizophrenia.

## Conclusion

In summary, our MR analysis found a negative correlation between asparagine and schizophrenia, indicating that asparagine reduces the risk of schizophrenia. However, there is no potential causal relationship between schizophrenia and asparagine. This study provides new ideas for the early detection of schizophrenia in the clinical setting and offers new insights into the etiology and pathogenesis of schizophrenia. Nonetheless, additional research is required to elucidate the potential mechanisms that underlie the association between aspartate and asparagine with schizophrenia.

## Data Availability

The datasets generated and analysed during the current study are available in the GWAS repository. https://gwas.mrcieu.ac.uk/datasets/met-a-388/, https://gwas.mrcieu.ac.uk/datasets/met-a-638/, https://gwas.mrcieu.ac.uk/datasets/ieu-b-42/.
